# Isolation of Primary Mouse Pulmonary Microvascular Endothelial Cells and Generation of an Immortalized Cell Line to Obtain Sufficient Extracellular Vesicles

**DOI:** 10.3389/fimmu.2021.759176

**Published:** 2021-12-08

**Authors:** Xu Liu, Feiping Xia, Xiao Wu, Ying Tang, Lu Wang, Qin Sun, Ming Xue, Wei Chang, Ling Liu, Fengmei Guo, Yi Yang, Haibo Qiu

**Affiliations:** Jiangsu Provincial Key Laboratory of Critical Care Medicine, Department of Critical Care Medicine, Zhongda Hospital, School of Medicine, Southeast University, Nanjing, China

**Keywords:** pulmonary microvascular endothelial cells, immortalized cell line, extracellular vesicles, cell culture, pulmonary inflammation

## Abstract

Pulmonary microvascular endothelial cells (PMECs) and the extracellular vesicles (EVs) derived from PMECs participate in maintaining pulmonary homeostasis and mediating the inflammatory response. However, obtaining a high-purity population of PMECs and their EVs from mouse is still notoriously difficult. Herein we provide a method to isolate primary mouse PMECs (pMPMECs) and to transduce SV40 lentivirus into pMPMECs to establish an immortalized cell line (iMPMECs), which provides sufficient quantities of EVs for further studies. pMPMECs and iMPMECs can be identified using morphologic criteria, a phenotypic expression profile (*e*.*g*., CD31, CD144, *G. simplicifolia* lectin binding), and functional properties (*e*.*g*., Dil-acetylated low-density protein uptake, Matrigel angiogenesis). Furthermore, pMPMEC–EVs and iMPMEC–EVs can be identified and compared. The characteristics of pMPMEC–EVs and iMPMEC–EVs are ascertained by transmission electron microscopy, nanoparticle tracking analysis, and specific protein markers. iMPMECs produce far more EVs than pMPMECs, while their particle size distribution is similar. Our detailed protocol to isolate and immortalize MPMECs will provide researchers with an *in vitro* model to investigate the specific roles of EVs in pulmonary physiology and diseases.

## Introduction

Pulmonary microvascular endothelial cells (PMECs) are an integral part of the alveoli–capillary barrier and are involved in the gas exchange. They are distinct from macrovascular endothelial cells and play an important role in pulmonary homeostasis and inflammation (1). PMECs dysfunction caused by various pathogens (*e*.*g*., bacterial, coronavirus) or systemic inflammatory stimuli induces critical pathophysiological changes in various pulmonary diseases, such as severe pneumonia, acute respiratory distress syndrome (ARDS), sepsis-induced acute lung injury, and chronic obstructive pulmonary disease (COPD) (2).

Given the important role of PMECs, culturing PMECs *in vitro* is essential to elucidate the molecular and cellular mechanisms involved in pulmonary diseases. Studies involving primary mouse pulmonary microvascular endothelial cells (pMPMECs) have been limited by the difficulties in acquiring these cells in culture (3). Although respectable isolation methods have been published, consistent results are difficult to produce by these strategies (4–6). The most common approach for isolating pMPMECs involves the enzymatic digestion of lung tissues and the immunoabsorption of pMPMECs to CD31 or CD102 magnetic beads. However, in our experiences, the positive selection of pMPMECs results in a small number of cells, low proliferative capacity, and early senescence. Furthermore, non-specific binding of magnetic beads often results in contamination by other cells present in the lung tissues, such as fibroblast, smooth muscle cells, epithelial cells, and leukocytes.

Recently, PMEC-derived extracellular vehicles (PMEC–EVs) have been of particular interest as they are secreted by PMECs directly into the bloodstream and can interact with circulating leukocytes to modulate local immune reactions (7). An increasing number of studies have demonstrated that endothelial cell-derived EVs are associated with the pathogenesis of inflammatory pulmonary diseases (8–10), but the specific roles and characteristics of PMEC–EVs in mediating pulmonary diseases are just beginning to be explored. Owing to the limited ability of pMPMECs to secrete EVs and the absence of a reliable cell line of pMPMECs, many researchers have used umbilical vein endothelial cell-derived EVs instead of PMEC–EVs in their studies. However, EVs present a high heterogeneity, and their cargos vary greatly depending on different sources. Thus, using other endothelial cell-derived EVs to explore the role of PMEC–EVs in pulmonary diseases may lead to a research bias.

Herein we provide a protocol that we established to harvest and culture high-purity populations of pMPMECs. Briefly, after dispersing the lung rims into a single-cell suspension, pMPMECs are enriched by depleting leukocytes, fibroblasts, and epithelial cells using anti-CD45 (11), anti-CD90.2 (12, 13), and anti-CD326 (14) magnetic beads individually. pMPMECs are cultured and then purified by using cloning cylinders. Moreover, we successfully establish immortalized mouse pulmonary microvascular endothelial cells (iMPMECs) with lentivirus SV40, making it possible to obtain abundant EVs *in vitro*. The sources, purity, and characteristics of cultured pMPMECs and iMPMECs are evaluated using morphological criteria by phase-contrast microscopy, phenotypic expression profile (CD31, CD144) (15), lectin binding, and endothelial function assays such as Dil-acetylated low-density lipoprotein (Dil-AcLDL) uptake, tube formation on Matrigel, and inflammatory responses *in vitro*. The characteristics of isolated iMPMEC–EVs can be identified and compared with pMPMEC–EVs by electron microscopy, nanoparticle tracking analysis, and the detection of specific protein markers of the EVs.

## Materials and Equipment

- DMEM-F12 (Gibco, cat. no. C11765500BT)
- Endothelial cell growth supplement (ECGS) (ScienCell, cat. no. 1052)
- Heparin (Sigma-Aldrich, cat. no. H3149)
- D-valine (Sigma-Aldrich, cat. no. V1255)
- Fetal bovine serum (FBS; Gibco, cat. no. 10099141)
- Antibiotic–antimycotic (Gibco, cat. no. 15240062)
- Phosphate-buffered saline (PBS, without calcium and magnesium, pH 7.2, Gibco, cat. no. C20012500BT)
- Collagen type I solution (Solarbio, cat. no. C8062)
- Glacial acetic acid (Sigma-Aldrich, cat. no. A6283)
- Ethanol, 75% (v/v)
- Collagenase type I (Sigma-Aldrich, cat. no. C0130)
- 10X Red blood lysing buffer (BioGems, cat. no. 64010-00)
- TrypLE EXPRESS (Gibco, cat. no. 12605028)
- EDTA (Sigma-Aldrich, cat. no. E8008)
- EasySep Mouse CD45 Positive Selection Kit (STEMCELL, cat. no. 18945)
- EasySep Mouse CD90.2 Positive Selection Kit (STEMCELL, cat. no. 18951)
- CD326 (EpCAM) MicroBeads (Miltenyi Biotec, cat. no. 130-105-958)
- GFP lentivirus control vector (abm, cat. no. LV095)
- Lenti-SV40 virus, high titer (abm, cat. no. G203)
- Lenti-SV40T (Puro) virus (abm, cat. no. G256)
- Lenti-human telomerase reverse transcriptase (hTERT) virus (abm, cat. no. G200)
- Lenti-human papillomavirus (HPV)-16 E6/E7 (Puro) virus (abm, cat. no. G268)
- Lenti-Ras V12 lentivirus (abm, cat. no. G221)
- ViralEntry transduction enhancer (abm, cat. no. G515)
- Vacuum grease, sterilized (Dow Corning, cat. no. L3484)
- GentleMACS Dissociators (Miltenyi Biotec)
- GentleMACS C tubes (Miltenyi Biotec, cat. no. 130-093-237)
- Cell scraper (Biologix, cat. no. 70-1180)
- Cell strainers, nylon (Biosharp, cat. no. BS-70-XBS, 70 μm; BS-40-XBS, 40 μm)
- 50-ml centrifuge tubes (Corning, cat. no. 430828)
- 15-ml centrifuge tubes (Corning, cat. no. 430791)
- 5-ml polystyrene round-bottom tube (STEMCELL, cat. no. 38007)
- 2-ml Eppendorf tubes, sterilized
- Cloning cylinder, sterile (Fisher Scientific, cat. no. 07-907-10)
- LS columns (Miltenyi Biotec, cat. no. 130-042-401)
- MACS MultiStand (Miltenyi Biotec, cat. no. 130-042-303)
- MidiMACS Separation Unit (Miltenyi Biotec, cat. no. 130-042-302)
- 24-well plates (BD Biosciences Falcon)
- T-75 culture flasks (Corning, cat. no. 430641)
- Vacuum filtration system, 0.22-μm (Millipore, cat. no. SCGPU05PR)
- EasySep Magnet (STEMCELL, cat. no. 18000)
- Centrifuge with temperature regulator
- Anatomical scissors, sterilized
- Ophthalmic forceps, sterilized
- 6-cm petri dishes, sterile (Corning, cat. no. 430166)
- 20- and 1-ml syringes
- 14-G metal cannula, sterilized
- Assorted pipettes (2–20, 10–100, and 100–1000 μl) with sterile, disposable tips
- Inverted microscope
- Glass dish
- 37°C bath

### Preparation of Reagents

- Heparin solution: Dissolve 500,000 IU heparin powder in 40 ml sterile PBS and filter-sterilize the solution using a 0.22-μm vacuum filtration system. This solution can be stored at 4°C for up to 1 month.
- D-valine solution: Dissolve 1 g D-valine powder into 20 ml sterile PBS in 37°C water bath to a concentration of 50 mg/ml and filter-sterilize the solution with a 0.22-μm vacuum filtration system. This solution can be stored at -20°C for up to 1 month. The D-valine solution should be stored frozen in single-dose vials, and repeated freezing and thawing should be avoided.
- Basal DMEM-F12 1% (v/v) antibiotic-antimycotic, 100 IU/ml heparin and DMEM-F12. Store this medium at 4°C for up to 2 months.
- Complete DMEM-F12 1% (v/v) antibiotic–antimycotic and 5% (v/v) FBS and DMEM-F12. Store this medium at 4°C for up to 1 month.
- MPMECs culture medium 1% (v/v) antibiotic–antimycotic, 5% (v/v) FBS, 1% (v/v) ECGS, 100 IU/ml heparin, 92 mg/L D-valine, and DMEM-F12. Store at 4°C. This medium should be used up within 2 weeks.
- Digestive solution: Dissolve 100 mg of collagenase type I powder per milliliter of 1X PBS and filter-sterilize it with a 0.22-μm vacuum filtration system to make 10X collagenase type I solution and store the stock solution at -20°C. The digestive working solution is made up of 1 ml 10X collagenase type I stock solution and 9 ml complete DMEM-F12. Prepare the working solution fresh on the day of the experiment.
- 1X red cell lysis buffer: Add 1 ml 10X red cell lysis buffer to 9 ml deionized water and warm the solution to room temperature. Prepare the digestive solution fresh on the day of the experiment.
- Sorting buffer: 2% (v/v) FBS, 1 mM EDTA, and PBS. Store this buffer at 4°C for 1 week.
- Sterile vacuum grease: Spread a thin layer of vacuum grease on a glass dish. Place the lid and wrap the entire dish with aluminum foil. Autoclave the dish and allow it to cool naturally. Store it in a sterile place under 30°C for up to half a year.
- Coating flasks or plates with collagen type I: Dilute glacial acetic acid to a concentration of 0.36 g/L with sterile PBS and filter-sterilize it with 0.22-μm vacuum filtration. Dilute collagen type I stock solution with 0.36 g/L glacial acetic acid to a concentration of 12 ug/ml working solution. Use 300 ul/well of the collagen type I working solution to coat 24-well plates or 10 ml collagen type I working solution for coating T-75 flasks. Incubate the plates or T-75 flasks on the benchtop overnight at 25°C and aspirate the excess solution on the next day. Use PBS to wash the coated plates or flasks 2 to 3 times before seeding the cells. The coated plates or flasks can be stored at 4°C for 3 months.

### Methods

**Figure 1:**
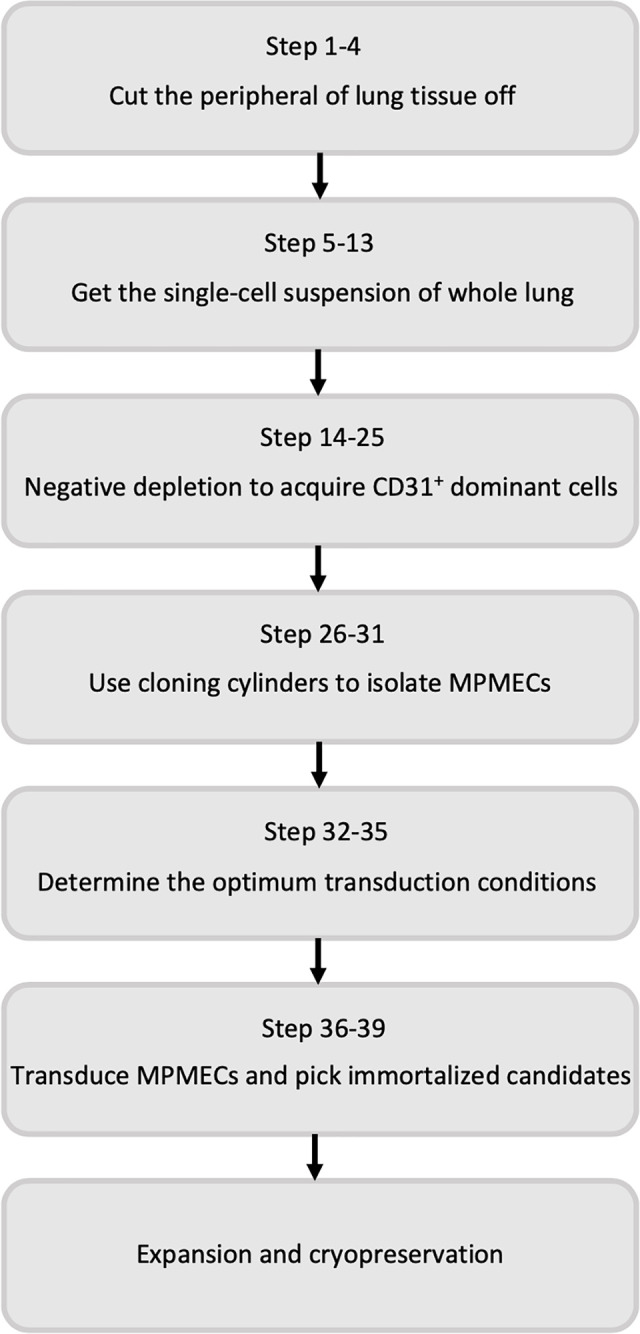
Flow diagram of the steps involved in primary mouse pulmonary microvascular endothelial cell isolation and immortalized mouse pulmonary microvascular endothelial cell generation.

**Figure 2:**
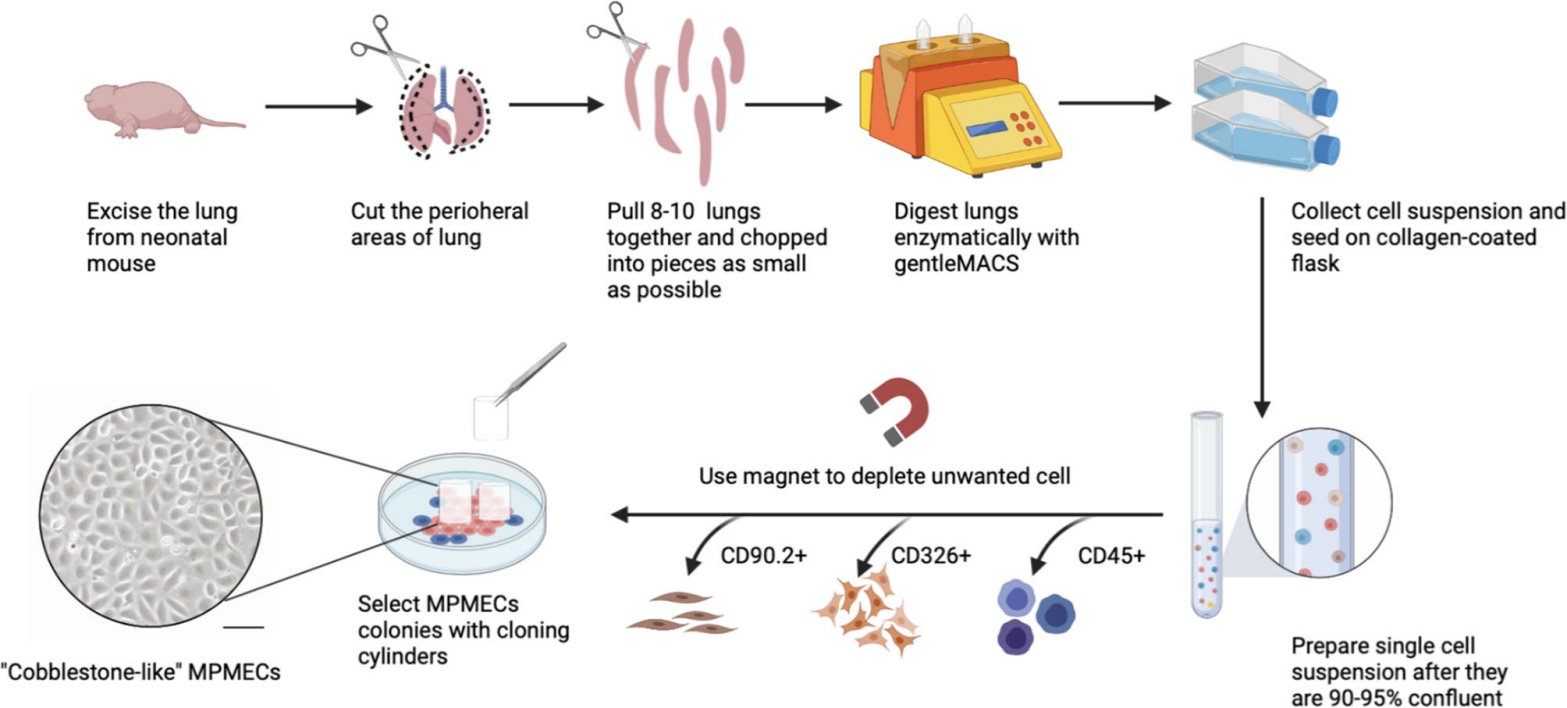
Schematic representation of primary mouse pulmonary microvascular endothelial cell (pMPMEC) isolation. Peripheral rims of mice lungs are chopped into pieces and digested into a single-cell suspension. The whole lung cells are plated in type I collagen-coated T-75 flasks. CD31^+^ predominant cells are chosen by depleting CD45^+^, CD90.2^+^, and CD326^+^ cells using magnetic beads. High-purity pMPMEC clones are carefully marked and isolated by using a cloning cylinder. pMPMECs are cultured in a specifically designed medium and prepared for lentivirus transduction. Scale bar, 50 μm. This figure is created *via* Biorender (biorender.com).

**Figure 3:**
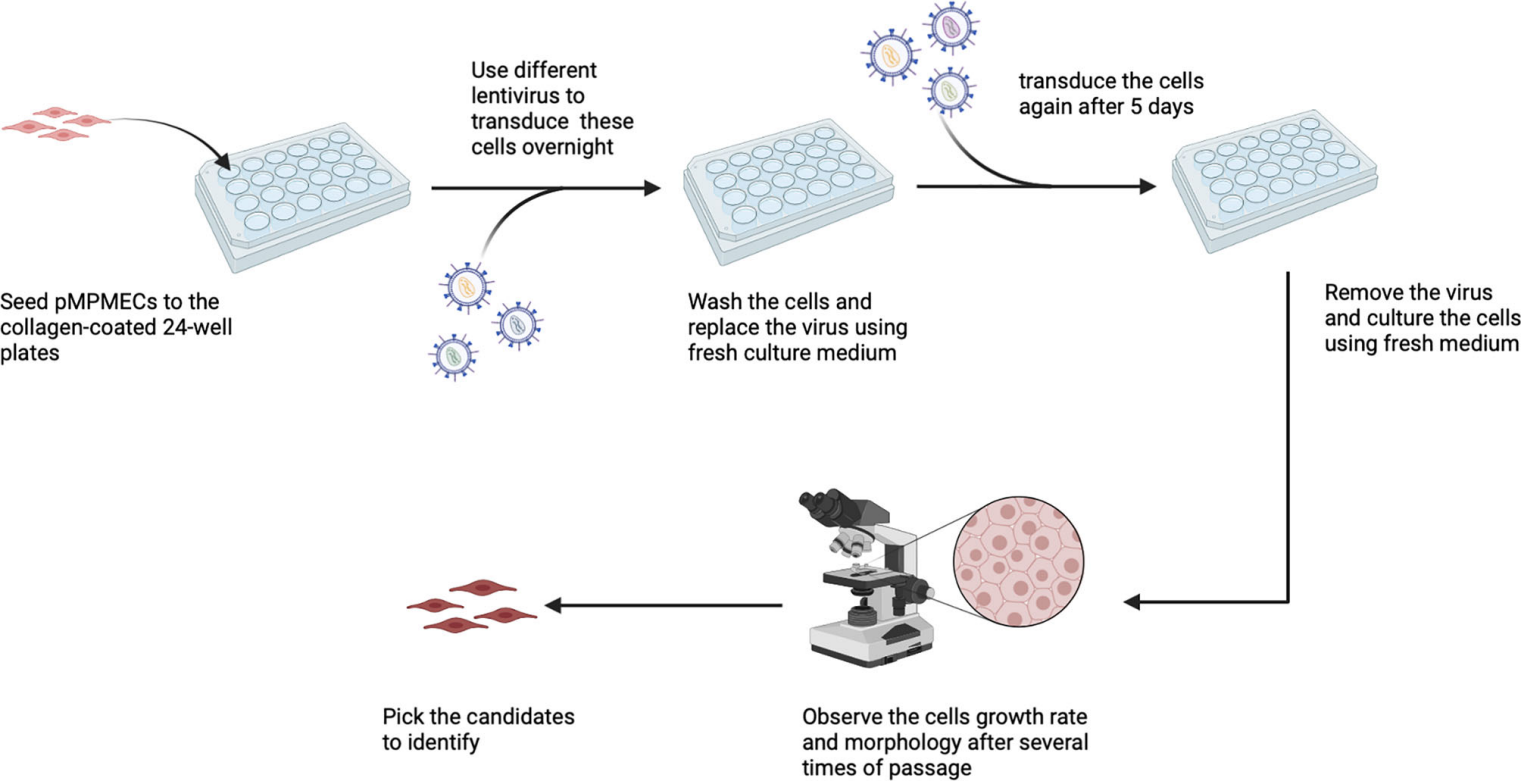
Schematic representation of mouse pulmonary microvascular endothelial cell (MPMEC) immortalization by lentiviruses. Different lentiviruses are used to transduce cells twice and observe the second-transduced MPMEC growth rate and morphology and choose the immortalized candidates. This figure is created *via* Biorender (biorender.com).

**Figure 4:**
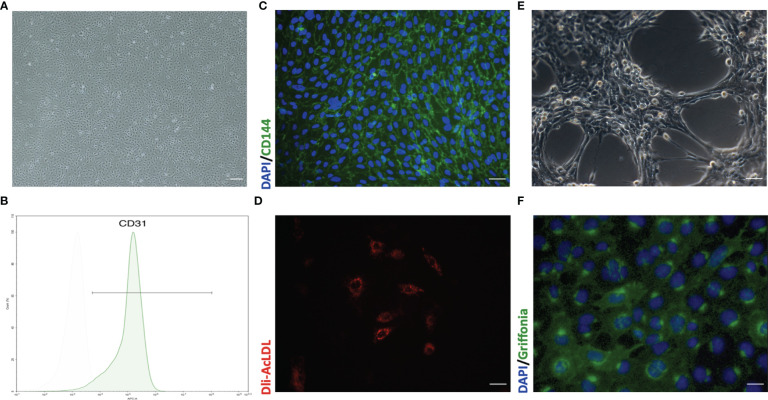
Representative morphology and identification of primary mouse pulmonary microvascular endothelial cells (pMPMECs). **(A)** pMPMECs form a “cobblestone-like” morphology when cultured as a monolayer. Scale bar, 200 μm. **(B)** Flow cytometry analysis of CD31 expression of pMPMECs. Cells are incubated directly with anti-CD31 APC-conjugated antibody for 30 min and washed twice before analyzing. The isolated mouse pulmonary microvascular endothelial cells show that over 94% cells are CD31-positive. **(C)** Representative immunofluorescence images of pMPMECs co-stained with VE-cadherin (green) (also known as CD144) and DAPI (blue). The cells are fixed, permeabilized, blocked, and then incubated with primary antibody at 4°C overnight. The cells are incubated with secondary antibody for 1 h at room temperature and counterstained with DAPI. Scale bar, 100 μm. **(D)** pMPMECs are incubated with Dil-AcLDL for 12 h Scale bar, 50 μm. **(E)** pMPMECs form a tube-like structure when cultured on Matrigel for 6 h Scale bar, 100 μm. **(F)** pMPMECs are stained with *Griffonia* lectin (green) after 1 h of incubation after fixation. Counterstaining with DAPI (blue) demonstrates that all cells are positive for lectin-binding. Scale bar, 50 μm.

**Figure 5:**
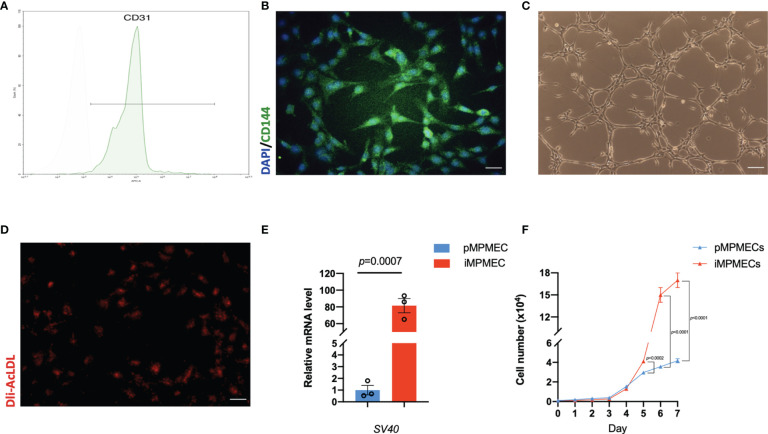
Representative phenotypic and functional analysis of immortalized mouse pulmonary microvascular endothelial cells (iMPMECs). **(A)** Flow cytometry analysis of iMPMECs. Over 97% cells are CD31-positive. **(B)** iMPMECs also express CD144 (green) counterstained with DAPI (blue). Scale bar, 100 μm. **(C)** iMPMECs can form an endothelial network on Matrigel. Scale bar, 100 μm. **(D)** iMPMECs can also uptake Dil-AcLDL (red) in co-cultures with it for 12 h Scale bar, 100 μm. **(E)** The SV40 mRNA expression of iMPMECs is measured by real-time PCR, n = 3. *p*-value is determined by Student’s *t*-test. **(F)** The cell proliferation rate of iMPMECs and primary mouse pulmonary microvascular endothelial cell is measured by cell counts, *n* = 6. *p*-values are determined by Student’s *t*-test.

A comprehensive overview of the step-by-step procedure for isolating pMPMECs is shown in **Figure 1**. After digestion of neonatal mouse peripheral lung tissue enzymatically, the cells obtained are seeded on collagen-coated T-75 flasks. CD31^+^ dominant cells are selected by depleting CD45^+^, CD326^+^, and CD90.2^+^ cells using a magnet and are plated on a petri dish in endothelial-specific growth conditions. The pMPMECs colonies form after approximately 6 to 7 days in culture and are carefully isolated using cloning cylinders and expanded to yield high-purity pMPMECs (**Figure 2**). pMPMECs are then seeded into collagen-coated 24-well plates and transduced by different lentiviruses twice. iMPMECs are selected based on the cell growth rate and morphology (**Figure 3**). The phenotype of pMPMECs and iMPMECs is confirmed by multiple experiments (**Figure 4** and **Figure 5**).

#### Preparation of Neonatal Mouse Lung Samples

1. Anesthetize mice with an intraperitoneal injection of 5% pentobarbital sodium (4 ml/kg).
2. Cover the entire body of the mice with 75% (v/v) ethanol for a few minutes.
3. Open the chest and remove the lungs with sterilized anatomical scissors. Transfer the lungs into a 6-cm petri dish with basal DMEM-F12 (as described above in the “Preparation of Reagents” section) and immediately place the dish on ice.
4. Remove the visceral pleura of the lung and dissect the peripheral tissues.

#### Acquisition of Single-Cell Suspension of Peripheral Lung Tissues

5. Transfer the peripheral lung tissues into a 5-ml Eppendorf tube and wash the tissues with basal DMEM-F12 three times.
6. Add 2 ml digestive solution (as described in “Preparation of Reagents” section) and mince the tissues into small pieces using sterilized ophthalmic scissors. Transfer the pieces in digestive solution to a gentleMACS C tube and add another 8 ml of freshly prepared digestive solution. Position the gentleMACS C tube on the gentleMACS Dissociators and run the “m-lung-01-02” program.
7. Transfer the gentleMACS C tube into a constant-temperature incubator shaker and continue digesting the peripheral lung tissue sections at 37°C for 30 min under horizontal shaking (200 rpm).
8. Place the gentleMACS C tube on the gentleMACS Dissociators and run the “m-lung-02.1” program. Next, add an equal volume of complete DMEM-F12 (as described in the “Preparation of reagents” sections) to quench the digestive solution.
9. Pipet the cell mixture up and down 20 times using a sterilized 14-G metal cannula attached to a disposable 20-ml syringe and filter the cell suspension through a 70-μm cell strainer. Wash the cell strainer with complete DMEM-F12 (2 ml/time, 3 times).
10. Centrifuge the cell mixture at 400 ×*g* at 4°C for 5 min and discard the supernatant; resuspend the whole cell pellets with 10 ml complete DMEM-F12 and centrifuge the cell mixture again at 400 ×*g* at 4 °C for 5 min.
11. Discard the supernatant and add 2 ml 1X red cell lysis buffer (as described in the “Preparation of Reagents” section). Pipet the cells up and down several times gently and let them rest at room temperature for 5 min. Then add a 10-times volume of complete DMEM-F12 to dilute the red cell lysis buffer.
12. Filter the cell suspension through a 40-μm cell strainer, wash the cell strainer with complete DMEM-F12 (2 ml/time, 3 times), and centrifuge the cell mixture at 400 ×*g* at 4°C for 5 min.
13. Discard the supernatant and add fresh MPMECs culture medium (as described in the “Preparation of Reagents” section) to obtain a cell suspension of 10^7^ cells/ml. Pipet the cells up and down several times.

#### Preparation of Cell Suspension Before Negative Magnetic Depletion

14. Add 6–8 ml fresh MPMEC culture medium to a flask and add the cell suspension; mix well and place the flasks in a humidified incubator (37°C, 5% CO_2_) for 24 h.
15. Remove the culture medium slowly with an electronic pipette and replace with 6–8 ml fresh MPMEC culture medium added slowly to the flask and return it to the incubator. Change the medium every 2 days. Use basal DMEM-F12 to wash the cells 2 to 3 times before refreshing the medium.
16. Use TrypLE Express to detach cells when they are 90–95% confluent (4). Remove the culture medium and wash the cells 2 to 3 times. Add 5 to 6 ml TrypLE Express and incubate the flask for 5 min at room temperature. Then, add the same volume of MPMEC culture medium to the flask to dilute TrypLE Express. Observe the cells on an inverted microscope. If some cells remain attached to the flask, use a sterile cell scraper to help detach them.
17. Collect the entire volume into a 15-ml tube and centrifuge the mixture at 400 ×*g* at 4°C for 5 min.
18. Discard the supernatant and add sorting buffer (as described in the “Preparation of Reagents” section) to resuspend the cells.

#### Depletion of CD45^+^, CD90.2^+^, and CD326^+^ Cells

19. Adjust the cell suspension to the recommended concentration according to the recommendation of the manufacturer.
20. Incubate the cells with the CD45-positive selection mixture for 5 min at room temperature. Then, add RapidSpheres into the tube and incubate for an additional 3 min. Top the volume up to 2.5 ml and deplete the CD45^+^ cells using the Easysep magnet.
21. Transfer the supernatant into a fresh 15-ml tube and centrifuge it at 400 ×*g* at 4°C for 5 min. Discard the cell-free supernatant and adjust the CD45^-^ cell pallet to the concentration recommended by the manufacturer.
22. Incubate the CD45^-^ cells with CD90.2-positive selection mixture for 5 min at room temperature. Then, add RapidSpheres into the tube and incubate them for an additional 3 min. Top the volume up to 2.5 ml and deplete the CD90.2^+^ cells using the Easysep magnet.
23. Transfer the supernatant into a fresh 15-ml tube and centrifuge it at 400 ×*g* at 4°C for 5 min. Discard the cell-free supernatant and adjust the CD45^-^CD90.2^-^ cell pallet to the recommended concentration.
24. Mix the CD45^-^CD90.2^-^ cell with CD326 MicroBeads at 4°C for 15 min with gentle tilting and rotation. Deplete the CD326^+^ cells using a LS magnetic column.
25. Collect the flow-through and centrifuge it at 400 ×*g* at 4°C for 5 min. Discard the supernatant and add some MPMEC culture medium to resuspend the CD45^-^CD90.2^-^CD326^-^ cells.

#### Isolation of pMPMECs

26. Seed 10^6^ cells on one 6-cm collagen-coated petri dish and add 4 ml fresh MPMEC culture medium and mix well.
27. Place the petri dish in a humidified incubator (37°C, 5% CO_2_) and refresh the medium every 2 days. Observe the cells daily with an inverted microscope and outline the boundaries of the MPMEC colonies with a black marker on the underside of the petri dish.
28. Use sterilized ophthalmic forceps to pick up cloning cylinders and put the bottom surface of the cloning cylinder onto autoclaved vacuum grease gently to create a uniform film.
29. Aspirate the culture medium and wash the cells with basal DMEM-F12 (3 ml/time, 3 times). Then, pick up the coated cloning cylinders upon the colony boundaries drawn before and press the cylinders tightly onto the petri dish surface using the ophthalmic forceps.
30. Add 100 μl TrypLE Express to each cloning ring with a 10–100-μl pipette and incubate the dish for 5 min. Then, add 100 μl MPMEC culture medium to dilute the TrypLE Express and pipette up and down gently to help in detaching the cells.
31. Remove the entire volume into a 2-ml Eppendorf tube and pool 6 to 8 cloning rings together. Centrifuge the whole volume at 400 ×*g* at 4°C for 5 min. Add 400 μl MPMEC culture medium to resuspend the cells up and seed the cells into collagen-coated 24-well plates and culture them in a humidified incubator (37°C, 5% CO_2_).

#### Determine the Optimal Transduction Reagents and Conditions

32. Seed pMPMECs in 500 μl of culture medium into a collagen-coated 24-well plate (3 × 10^4^ cells/well in 7 wells) and culture for 24 h.
33. Thaw the lentivirus-harboring GFP only (1 × 10^7^ IU/ml) in 37°C water bath and remove it from the bath immediately after thawing. Then, add 30, 60, 90, 120, and 150 μl lentivirus stock solution containing 5 μl ViralEntry to each well to establish different values of multiplicity of infection (MOI = 10, 20, 30, 40, 50). Leave one well free of lentivirus. Adjust the volume of each well to 500 μl using the MPMEC culture medium.
34. Centrifuge the 24-well plate at 4,000 rpm for 30 min to increase the transduction efficiency. Then, incubate the cells for 72 h in a humidified incubator (37°C, 5% CO_2_).
35. Use fluorescence microscopy to take pictures of cells in different wells after 36, 48, 60, and 72 h. Determine the transduction rate per well at different MOIs and different time intervals.

#### Immortalization of pMPMECs

36. Seed pMPMECs in 500 μl fresh MPMEC culture medium into collagen-coated 24-well plates (3 × 10^4^ cells/well) and culture for 24 h.
37. haw the lentiviruses [Lenti-SV40, Lenti-SV40T, Lenti-SV40 T (puro), Lenti-Ras V12, Lenti-hTERT, and Lenti-HPV-16 E6/E7 (puro)] in 37°C water bath and remove them from the bath immediately after thawing. Next, add the appropriate volume of lentivirus particles into the MPMEC culture medium containing 5 μl ViralEntry to achieve an optimal MOI (in our experiments, the optimal MOI to transduce the MPMECs was 30).
38. Aspirate the culture medium and incubate the cells with the viral supernatant in different wells in a humidified incubator and exchange the viral supernatant with fresh MPMEC culture medium after 48 h.
39. Use TrypLE Express to detach the cells when they are 90–95% confluent. Reseed the first-transduced pMPMECs in 500-μl culture medium on collagen-coated 24-well plates (3 × 10^4^ cells/well). Change the culture medium every 2 days. Repeat steps 36–38 to repeat the transduction of pMPMECs and monitor the cell growth rate and morphology so as to select the immortalized candidates.

## Results

### Identification of pMPMECs

pMPMECs form cobblestone-like colonies when cultured as a monolayer on type I collagen-coated plates (**Figure 4A**). Two cell surface CD markers are used to identify the pMPMECs using FACS analysis, which indicates that over 94% of the cells express CD31 (**Figure 4B**), and the immunofluorescence analysis shows a near-complete positivity for CD144 (**Figure 4C**). pMPMECs exhibit the engulfing capacity of Dil-AcLDL (**Figure 4D**), and pMPMECs grown on Matrigel can form tubular-like structures (**Figure 4E**). The pMPMECs are positive to bind *G. simplicifolia* lectin (**Figure 4F**), which indicates that they derive from the pulmonary microvascular endothelium rather than from the extra-alveolar endothelium.

### Morphology of MPMECs Transduced by Different Lentiviruses

**Figure 6:**
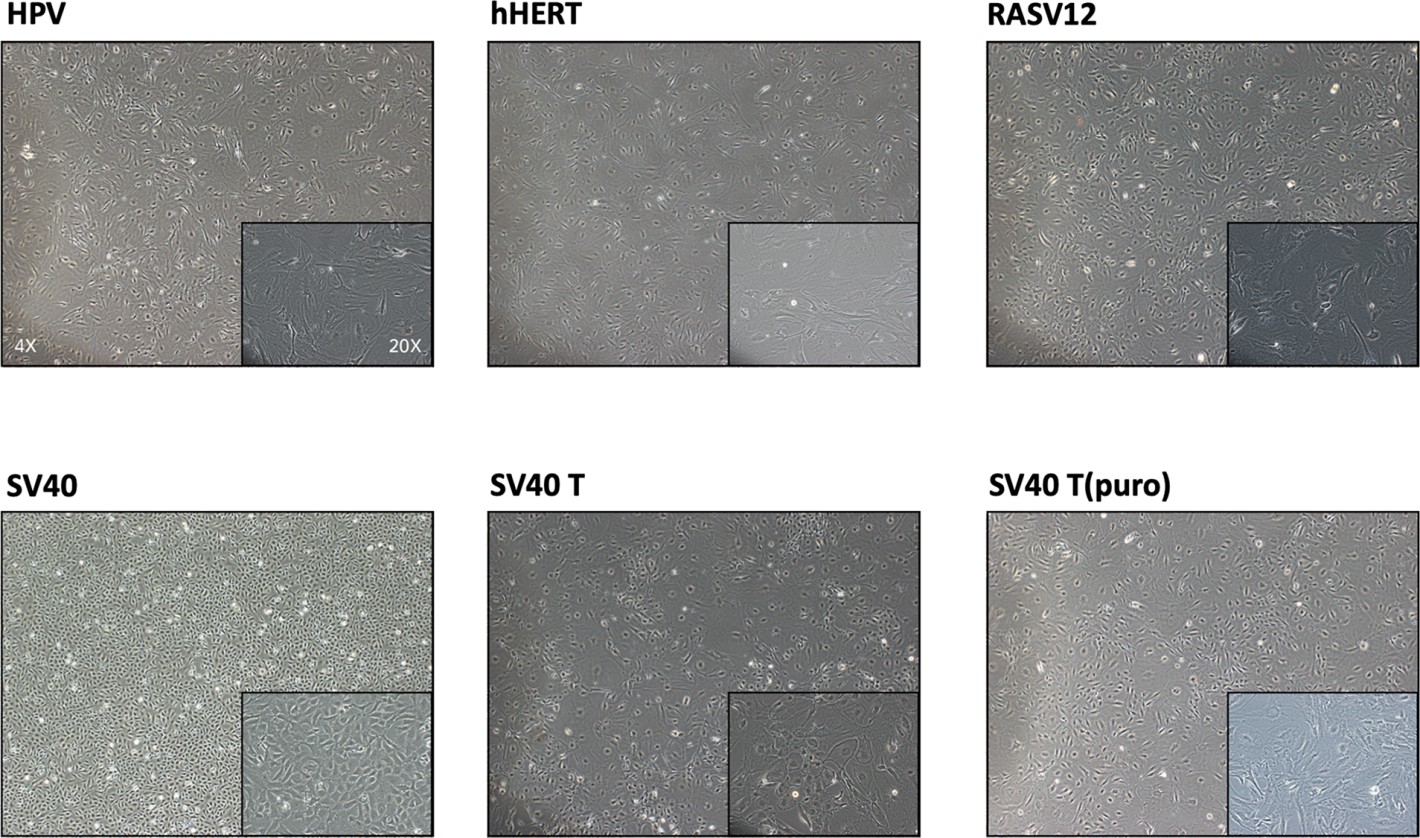
Morphology of mouse pulmonary microvascular endothelial cells (MPMECs) transduced by different lentiviruses. Illustration of the second transduction of MPMECs using different lentiviruses (brightfield phase-contrast images). Only the Lenti-SV40 group can maintain the “cobblestone-like” morphology of endothelial cells when cultured as a monolayer and proliferate stably.

Transduced pMPMECs are cultured on 24-well plates and passaged when they are 90–95% confluent. Only cells transduced by lenti-SV40 can grow well and maintain a uniform morphology after 10 passages. Cells transduced by other lentiviruses display typical signs of senescence, *e*.*g*., flat, large, binucleation, and granulation, and are even prone to endothelial–mesenchymal transition (**Figure 6**). Only lenti-SV40-transduced MPMECs have extended proliferation beyond pMPMECs and become immortalized.

### Identification of iMPMECs

iMPMECs are subjected to FACS analysis for the expression of endothelium-specific markers. CD31 staining is positive in 97% of the cells (**Figure 5A**). Almost all cells are stained immunofluorescently for CD144 (**Figure 5B**). Tube/cordlike structures are also observed when the iMPMECs are cultured on Matrigel (**Figure 5C**). The Dil-AcLDL uptake assay indicates that iMPMECs maintain this capacity of pMPMECs (**Figure 5D**). Total RNA of pMPMECs and iMPMECs are extracted, and cDNA are synthesized to real-time PCR with SV40-specific primers to detect its expression. The SV40 transgene is not detected in pMPMECs but is detected in iMPMECs, indicating the successful immortalization of pMPMECs (**Figure 5E**). The proliferation rates of pMPMECs and iMPMECs are determined by absolute cell counts each day from day 1 to day 7. The pMPMECs and iMPMECs are seeded at a density of 1 × 10^3^ cells/well in 96-well dishes and counted every 24 h using a hemocytometer. This assay indicates that iMPMECs have a much greater proliferative rate than pMPMECs (**Figure 5F**).

### Inflammatory Responses of pMPMECs and iMPMECs

**Figure 7:**
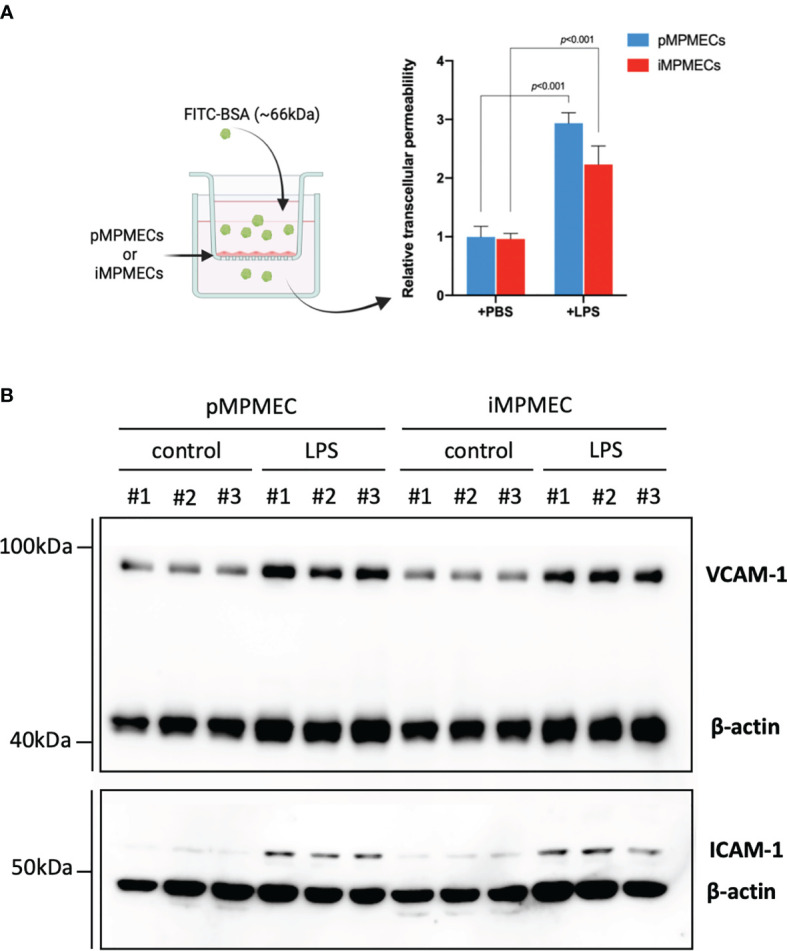
Immortalized mouse pulmonary microvascular endothelial cell (iMPMEC) function and response to inflammatory treatment. **(A)** The permeability of lipopolysaccharide (LPS)-treated primary mouse pulmonary microvascular endothelial cells (pMPMECs) and iMPMECs monolayers grown on 0.4-μm filters is measured by the passage of fluorescein isothiocyanate–bovine serum albumin probes added to the top well at the beginning of the experiment. The absorbance at 550 nm at 24 h later is measured in the top and bottom wells, *n* = 3. The *p*-values shown are determined by Student’s *t*-test. **(B)** Western blotting analysis to detect the inflammatory-related protein VCAM-1 and ICAM-1 expression of LPS-treated pMPMECs and iMPMECs. Cells are collected, lysed, and denatured with loading buffer. The samples were loaded into FuturePAGE gel, and the gel was run as recommended by the manufacturer. The proteins in the gel are transferred to polyvinylidene fluoride membranes. The membranes are blocked with 5% nonfat dry milk at room temperature for 2 h and then incubated with primary antibodies at 4°C overnight. On the next day, the membranes were washed with 1X Tris-buffered saline with 0.1% Tween^®^ 20 detergent for three times and then incubated with secondary antibody at room temperature for 1 h. The membranes were washed, and images of the enhanced chemiluminescence substrate were acquired; *n* = 3.

Endothelial cells can be the main target for pathogens or stimuli and may serve as an initiator for inflammatory response. Activated endothelial cells can secrete many mediators and trigger leukocyte adhesion and migration (1, 7, 16). The permeability assay shows that treatment of both pMPMECs and iMPMECs with lipopolysaccharide (LPS; 1 ug/ml for 24 h) can induce the passage of the fluorescent probes (FITC-BSA) from the top wells to the bottom wells (**Figure 7A**). Meanwhile, both pMPMECs and iMPMECs upregulate VCAM-1 and ICAM-1 expression (**Figure 7B**). The similar inflammatory responses of pMPMECs and iMPMECs to LPS treatment indicate that iMPMECs can replace pMPMECs in the study of endothelial functions and cytopathogenic effects in inflammatory pulmonary disease, such as ARDS.

### Characteristics of EVs Secreted by pMPMECs and iMPMECs

**Figure 8:**
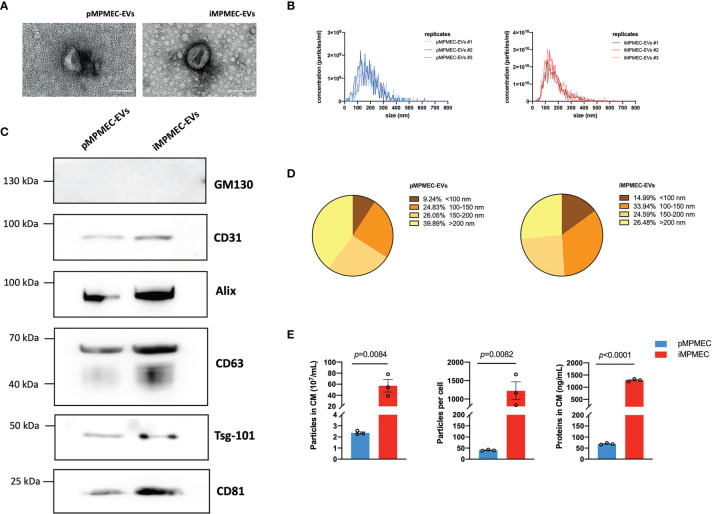
Generation and characterization of extracellular vesicles (EVs) secreted by primary mouse pulmonary microvascular endothelial cells (pMPMECs) and immortalized mouse pulmonary microvascular endothelial cells (iMPMECs). **(A)** Transmission electron microscopic images of EVs secreted by pMPMECs and iMPMECs. Scale bar, 200 nm. **(B)** Nanoparticle tracking analysis to show the size distribution and concentration, *n* = 3. **(C)** Western blotting analysis to show the positive and negative biomarkers of EVs. CD31 is used as a positive control of EVs derived from endothelial cells. **(D, E)** Quantification of EVs derived from pMPMECs and iMPMECs, *n* = 3. *p*-values are determined by Student’s *t*-test.

Lung microvascular endothelial cell-derived EVs serve as messengers to mediate an inflammatory immune response. Thus, it is of great significance to study the specific role of lung microvascular endothelial cell-derived EVs in inflammatory pulmonary disease. The EVs are isolated from the culture medium using centrifugation/ultracentrifugation according to the MISEV 2018 guidelines. After the pMPMECs or iMPMECs reach 80–85% confluence, the culture medium is exchanged with EV-free medium for 36 h. The supernatant is collected and centrifuged at 300 ×*g* for 5 min at 4°C to remove cells and debris. Next, the supernatant is collected and centrifuged at 2,000 ×*g* for 40 min at 4°C to remove apoptotic bodies. The supernatant is transferred to a glass tube to be ultracentrifuged at 120,000 ×*g* for 2 h at 4°C. The pellet is resuspended with PBS, followed by re-ultracentrifugation at the same parameters to wash the EVs. The tube lid is left open to air-dry the pellet at room temperature for 10 min, and the precipitation is resuspended, using 100 μl PBS, for subsequent analyses. iMPMECs can secrete EVs with similar appearance (**Figure 8A**), similar average particle size (**Figure 8B**), and similar particle size distribution (**Figure 8D**) as pMPMECs. Western blotting analyses reveal that both pMPMEC–EVs and iMPMEC–EVs express EV protein-positive markers, including CD63, CD81, ALIX, tumor susceptibility gene-101, and endothelium protein marker CD31, but do not express EV protein-negative marker GM130. (**Figure 8C**). The concentration of pMPMEC–EVs is 2.36 × 10^7^ ± 0.16 × 10^7^ particles per milliliter in the conditioned medium (CM) and 40 ± 2 particles per cell, and the concentration of iMPMEC–EVs is 57.2 × 10^7^ ± 19.62 × 10^7^ particles per milliliter in CM and 1,222 ± 419 particles per cell. Besides these, the mean protein concentration of pMPMEC–EVs is 69.15 ± 3.76 ng/ml, and the mean protein concentration of iMPMEC–EVs is 1,291.30 ± 52.93 ng/ml. Furthermore, pMPMECs possess a limited lifespan in long-term culture, and the ability of pMPMECs to secrete EVs is much poorer than iMPMECs (**Figure 8E**). Therefore, iMPMECs can be a good substitute for pMPMECs to generate EVs *in vitro*.

## Discussion

Studies involving cultured pMPMECs or MPMEC–EVs have been limited because of the difficulty in acquiring high-purity, high-viability cells and collecting a suitable amount of EVs. Herein we provide a novel and repeatable protocol to isolate pMPMECs and immortalize them with lentivirus SV40 to extend the proliferation lifespan so that they can secrete EVs continuously and stably. Ideal immortalized cells are not only capable of an extended lifespan but also possess an identical phenotype to their parental cells (17). Many morphological, molecular, and biochemical properties and assays are used to identify pMPMECs and iMPMECs, such as the cobblestone appearance (a typical morphology of confluent endothelial cells), tube-like structures on Matrigel, Dil-AcLDL protein uptake (a characteristic distinguishing from fibroblasts, smooth muscle cells, pericytes, and other stromal cells) (18), and phenotypic markers (CD31 and CD144). Lectin binding is an effective method to distinguish macro- and microvascular endothelial cells. *G. simplicifolia* lectins have been used to identify endothelium in mouse tissues, and our data shows that our isolated cell cultures derive from microvascular tissue (19). iMPMECs secrete nearly 20-fold EVs than pMPMECs, indicating that iMPMECs can be a good candidate to study pulmonary microvascular endothelial cell-derived EVs *in vitro*.

Several strategies for isolating pMPMECs have been reported in the literature. Most researchers claim that they could obtain enough high-purity pMPMECs by single [anti-CD31 (4) or anti-CD102 (20)] or double magnetic beads sorting [anti-CD31+ anti-CD102 (21), anti-CD102 twice (22), or anti-CD31 twice (23)] directly. Despite the effectiveness that they reported, we find that the use of magnetic beads to isolate pMPMECs results in very low cell yields, and contaminated cells are still existing. Besides these, the viability of sorted pMPMECs is often poor. More importantly, beads binding to the pMPMECs cannot be dissociated (24). These beads will generate shear stress in different directions and may mediate antibody-induced intracellular signaling, which influences cell homeostasis and function (25). In contrast to these commonly used methods, we propose a negative selection protocol of pMPMECs to avoid the problems mentioned above. Fibroblasts and other contaminated cells are isolated by immunoabsorption directly, and pMPMECs are free of magnetic beads to avoid any injury or antibody-mediated activation. To our knowledge, this is the first report to use this method to isolate pMPMECs.

In our protocol, leukocytes, fibroblasts, and epithelial cells are depleted by anti-CD45, anti-CD90.2, and anti-CD326 antibody conjugated magnetic beads in separate steps. CD45 (lymphocyte common antigen) is a receptor-linked protein tyrosine phosphatase and is expressed on all leukocytes. CD90.2 (Thy 1) antigen is a GPI-linked glycoprotein member of the immunoglobulin super family and is expressed on mesenchymal stem cells and fibroblasts (26). CD326, also known as epithelial cell adhesion molecule, is a 40-kDa type I transmembrane glycoprotein that is involved in modulating intercellular adhesion and is strongly expressed on various epithelial cells. The remaining cells are CD31^+^ dominant cells and are cultured in an optimally designed medium to support pMPMECs growth. The medium used contains several important components, including endothelial cell growth supplement, heparin, and D-valine. The endothelial cell growth supplement can provide a defined and balanced environment and maximally promote the growth of pMPMECs. Heparin can affect the mid-G1 phase of the cell cycle and thus inhibits vascular smooth muscle cell hyperproliferation (27). In addition, heparin exerts an additional protective effect on pMPMECs during cell culture (28). The protective mechanisms are related to the interaction of the histone methylation region and the regulation of multiple intercellular signal pathways.

In our experience, the removal of contaminating fibroblasts is very difficult even when using CD90.2 magnetic beads. Fibroblasts grow faster than pMPMECs and can become dominant and substitute pMPMECs. D-valine is an enantiomer of L-valine and has been widely used in cell culture as a selective inhibitor of fibroblast proliferation as these lack D-amino acid oxidase enzyme activity while showing no influence on pMPMECs growth (13, 29). To increase the purity of the pMPMECs, we use cloning cylinders to isolate pMPMEC colonies and pool them together to expand them in culture. pMPMECs display a cobblestone appearance when they reach confluency, and the boundaries are easily outlined with a fine-tipped marker. Encircling the cobblestone cell colonies with small cloning cylinders is very easy, and pMPMECs isolated in this way have high viability.

It is well known that primary cells have a very finite number of cell divisions and enter into replicative senescence quickly. pMPMECs are no exception, which limits their use in the study of inflammatory pulmonary diseases. To obtain a consistent number of cells to study *in vitro*, we choose to immortalize pMPMECs using viral genes. Lentiviruses can integrate genetic information into the DNA of host cells and manipulate their physiological function (30). Several lentiviruses have been applied for cell immortalization, such as simian virus 40 large T antigen (SV40-T), SV40, hTERT, and HPV. Different cell types present distinct sensitivity to different lentiviruses, for example, the over-expression of hTERT in some cell types (especially epithelial cells) can induce cell death and fails to immortalize cells (31). In our study, we use six commonly used lentiviruses to transduce pMPMECs, and only the cells transduced with SV40 can achieve an expanded lifespan while maintaining their morphology. SV40 is a small DNA tumor virus and has been widely used to transform a variety of cell types, including human vascular endothelium (32) and mouse lymphoid endothelial cells (33). SV40 encodes two tumor-associated antigens simultaneously: large T antigen and small T antigen (34). SV40 large T antigen has the potential to immortalize cells in most cases but has failed to transform pMPMECs. Small T antigen can help large T antigen to transduce cells in some specific circumstances (35). Immortalized MPMECs express SV40 gene stably and acquire a self-proliferating capacity while maintaining the state and function as pMPMECs.

Patients with severe pneumonia are often susceptible to ARDS and septic shock. PMECs are both participants and modulators of the pulmonary inflammatory process. They surround the alveoli and are targets of LPS present in the alveolar cavity or in the pulmonary circulation (2, 7, 36). PMECs express toll-like receptor 4 (TLR4) which has been identified as the primary receptor for LPS. LPS can stimulate TLR4 and modify the endothelial barrier function, promote hyperpermeability, and activate inflammatory cell infiltration (37). Following treatment (1 μg/ml, 24 h), LPS elicits a significant increase in pMPMECs and iMPMEC permeability consistent with known barrier disruption properties. In addition, the expression of VCAM-1 and ICAM-1 increased in pMPMECs and iMPMECs after LPS stimulation. VCAM-1 and ICAM-1 are both cell adhesion molecules and facilitate leukocyte trans-endothelial migration. VCAM-1 and ICAM-1 are present continuously in low concentrations on the membranes of endothelial cells and are upregulated after inflammatory stimuli (38). Our findings suggest that iMPMEC can be used as an alternative model to study LPS responses in pMPMECs *in vitro*.

After activation, PMECs secrete multiple mediators to regulate the inflammatory process. Soluble factors (*e*.*g*., cytokines) are considered as classical mediators of PMECs; however, newly discovered EVs have attracted increasing attention (39). PMEC–EVs contain various bioactive cargo (*e*.*g*., nucleic acids, lipids, and proteins) and can target leukocytes to manipulate them within the local immune microenvironment in theory (40). The specific role of PMEC–EVs in pulmonary diseases needs to be investigated further. An increasing number of studies have demonstrated that endothelial-derived EVs are involved in diverse pulmonary diseases, including ARDS, sepsis-induced acute lung injury, idiopathic pulmonary fibrosis, cystic fibrosis, and COPD, but it is worth nothing that EVs present high heterogeneity, and their cargo varies greatly depending on different sources; thus, using other endothelial cell-derived EVs to explore their roles in pulmonary diseases may lead to a bias in research emphasis. It is notoriously difficult to collect sufficient EVs from primary cells *in vitro* because of their extremely finite proliferation rate and lifespan. Almost all *in vitro* studies used cell lines or stem cells to obtain EVs. In our study, we establish the cell line, iMPMECs, and they can secrete a significant amount of EVs in the culture medium compared to pMPMECs. Considering the cell source, iMPMEC may represent a better tool than other tissue-derived endothelial cells to investigate the role of pulmonary microvascular endothelial cell-derived EVs in pulmonary diseases.

In conclusion, we provide a feasible and reproducible protocol to isolate high-purity pMPMEC and establish a cell line iMPMECs to carry out large-scale studies using EVs *in vitro*.

## Data Availability Statement

The original contributions presented in the study are included in the article/supplementary material. Further inquiries can be directed to the corresponding author.

## Ethics Statement

This animal study was reviewed and approved by the Institutional Animal Care and Use Committee of Southeast University.

## Author Contributions

XL and FX contributed equally to this work and to data acquisition. XL contributed to the conception of the protocol and wrote the first version of the manuscript. All authors contributed to the article and approved the submitted version.

## Funding

This research was supported by grants from the National Natural Science Foundation of China (grant number: 81930058), the National Science and Technology Major Project of the Ministry of Science and Technology of China (2020YFC0843700), and the Jiangsu Provincial Special Program of Medical Science (BE2018743, BE2019749).

## Conflict of Interest

The authors declare that the research was conducted in the absence of any commercial or financial relationships that could be construed as a potential conflict of interest.

## Publisher’s Note

All claims expressed in this article are solely those of the authors and do not necessarily represent those of their affiliated organizations, or those of the publisher, the editors and the reviewers. Any product that may be evaluated in this article, or claim that may be made by its manufacturer, is not guaranteed or endorsed by the publisher.
